# Preschoolers’ Comprehension of Functional Metaphors

**DOI:** 10.1162/opmi_a_00152

**Published:** 2024-07-19

**Authors:** Rebecca Zhu, Mariel K. Goddu, Lily Zihui Zhu, Alison Gopnik

**Affiliations:** Department of Psychology, Stanford University, Stanford, CA; Centre for Advanced Study in the Humanities: Human Abilities, Berlin, Germany; Institut für Philosophie, Freie Universität Berlin, Berlin, Germany; Department of Philosophy, Stanford University, Stanford, CA; Department of Cognitive Science, Johns Hopkins University, Baltimore, MD; Department of Psychology, University of California - Berkeley, Berkeley, CA

**Keywords:** metaphor, relational reasoning, language acquisition, cognitive development

## Abstract

Previous work suggests that preschoolers often misunderstand metaphors. However, some recent studies demonstrate that preschoolers can represent abstract relations, suggesting that the cognitive foundations of metaphor comprehension may develop earlier than previously believed. The present experiments used novel paradigms to explore whether preschoolers (*N* = 200; 4–5 years; 100 males, 100 females; predominantly White) can understand metaphors based on abstract, functional similarities. In Experiment 1, preschoolers and adults (*N* = 64; 18–41 years; 25 males, 39 females; predominantly White) rated functional metaphors (e.g., “Roofs are hats”; “Tires are shoes”) as “smarter” than nonsense statements (e.g., “Boats are skirts”; “Pennies are sunglasses”) in a metalinguistic judgment task (*d* = .42 in preschoolers; *d* = 3.06 in adults). In Experiment 2, preschoolers preferred functional explanations (e.g., “Both keep you dry”) over perceptual explanations (e.g., “Both have pointy tops”) when interpreting functional metaphors (e.g., “Roofs are hats”) (*d* = .99). In Experiment 3, preschoolers preferred functional metaphors (e.g., “Roofs are hats”) over nonsense statements (e.g., “Roofs are scissors”) when prompted to select the “better” utterance (*d* = 1.25). Moreover, over a quarter of preschoolers in Experiment 1 and half of preschoolers in Experiment 3 explicitly articulated functional similarities when justifying their responses, and the performance of these subsets of children drove the success of the entire sample in both experiments. These findings demonstrate that preschoolers can understand metaphors based on abstract, functional similarities.

## INTRODUCTION

A metaphor is a figurative utterance that directly compares a concept from one domain to another concept in an unrelated domain. Metaphors are found in everyday speech (e.g., “I got lost in a sea of people”) as well as famous creative works (e.g., Shakespeare’s “If music be the food of love, play on”). Metaphors facilitate communication and provide frameworks for reasoning about abstract concepts (Camp, 2009), influencing attention, memory, and information processing (Thibodeau et al., 2017, 2019). Metaphors are also a force for creative change: they can facilitate the discovery of new scientific theories (Kuhn, 1993) and the creation of new word meanings (Bowdle & Gentner, 2005; Holyoak & Stamenkovíc, 2018).

While metaphors promote novel ways of thinking and reasoning (Thibodeau et al., 2017), they also pose unique language comprehension challenges. A fluent English-speaking adult understands that someone who is “lost in a sea of people” does not need a life vest. But interpreting this metaphoric statement may require additional cognitive capacities beyond those that enable us to understand utterances concerning someone who is literally “lost at sea” or “lost in a crowd”. What are these additional cognitive capacities, and when and how do they develop?

Researchers have posited multiple theories of metaphor comprehension that rely on different underlying cognitive mechanisms, such as relational reasoning (Gentner, 1988; Gentner & Clement, 1988; Holyoak, 2019), categorization (Glucksberg & Keysar, 1990; Glucksberg et al., 1997), and embodied conceptual mappings (Lakoff & Johnson, 1980; Thibodeau et al., 2019). Of these mechanisms, *relational reasoning*—the ability to attend to similarities between objects based on their shared abstract relations (e.g., “both same”) rather than on their shared superficial features (e.g., “both red”)—is widely argued to play a critical role (Bowdle & Gentner, 2005; Gentner, 1988; Holyoak & Stamenkovíc, 2018; Roberts & Kreuz, 1994; Wolff & Gentner, 2011). In this literature, participants’ preferences for reasoning using abstract relations versus perceptual features is often assessed using card-matching tasks (Christie & Gentner, 2010). For example, a participant might be given a flashcard (the “sample” card) depicting two dogs. If they choose to match the sample card with another card depicting a dog and a cat, their match is based on relatively surface-level, object-based similarities (i.e., matching “dog” with “dog”). By contrast, if they instead choose to match the sample card depicting two dogs with a flashcard showing two birds, their match is based on relational similarities (i.e., matching “same” with “same”).

Metaphor comprehension seems to rely on a parallel capacity. To understand a novel metaphor, a listener must identify the basis for an equivalence drawn between two objects that are not conventionally associated with each other. In many metaphors, this equivalence is grounded in a shared abstract similarity rather than in a shared featural similarity. For example, making sense of the utterance “Clouds are sponges” entails recognizing that both objects *hold water*, a property that is not readily perceived.

Many researchers have argued that children do not understand metaphors in an adult-like fashion until late in development, possibly not until adolescence (Demorest et al., 1983; Silberstein et al., 1982; Winner et al., 1976, 1980). Researchers have attributed this failure in part to their inability to notice abstract similarities between concepts. In one study, for example, participants were asked to complete sentences (e.g., “The volcano is…). Six-year-olds tended to select more surface-level completions (e.g., “a bright firetruck”) and adults tended to select less surface-level completions (e.g., “a very angry man”) (Silberstein et al., 1982). Thus, when prompted to complete metaphorical utterances, children seem to prefer metaphors that highlight perceptual similarities over those that highlight more abstract similarities. Similarly, Nippold and Sullivan (1987) found that preschoolers did not correctly interpret novel metaphors (e.g., understanding that “the purse was an Easter basket with no candy” meant that the purse lacked money, as opposed to other features like zippers or handles). Interestingly, children’s responses in these metaphor studies track closely with their performance on traditional relational reasoning tasks. In such tasks, 3- and 4-year-olds strongly prefer perceptual matches over relational matches (e.g., Christie & Gentner, 2010). Taken together, these results could be taken to suggest that a general difficulty in detecting and reasoning about abstract commonalities underlies children’s failures in both relational reasoning and metaphor production tasks.

However, there are two areas of research that suggest that children’s competence with metaphors may emerge earlier than predicted by the previous tasks. First, recent studies that use novel paradigms to investigate the development of relational reasoning suggest that the capacity to represent abstract relations is, in fact, present in preschoolers (Carstensen et al., 2019; Christie & Gentner, 2014; Goddu et al., 2020; Hochmann et al., 2017, Holyoak et al., 1984), toddlers (Walker et al., 2016; Walker & Gopnik, 2017), and even infants (Anderson et al., 2018; Hochmann et al., 2016). By their preschool years, children are not only able to represent basic abstract relations such as sameness or difference of identity (Carstensen et al., 2019; Christie & Gentner, 2014; Hochmann et al., 2017), but also relations based on other dimensions, such as size, number, and color (Goddu et al., 2020). This new work suggests that cognitive mechanisms relevant for metaphor comprehension might be in place much earlier than previously supposed, and that children might show earlier competence at metaphor comprehension given different experimental methods.

Second, several recent studies have demonstrated some early metaphor comprehension in preschoolers (Özçalişkan, 2005, 2007; Pouscoulous & Tomasello, 2020). For example, Pouscoulous and Tomasello (2020) showed that children as young as three years of age correctly interpreted perceptual metaphors. Children who heard perceptual metaphors (e.g., “The dog with the brown shoes”) successfully selected perceptually similar target toys (e.g., a dog with brown feet) over incorrect distractor toys (e.g., a dog with a brown bow). Similarly, Özçalişkan (2007) showed that four-year-olds correctly interpreted metaphors comparing motion and space. Children who heard motion-space metaphors (e.g., “The things that her mom wanted escaped from her mind”) successfully selected the correct interpretations of the metaphors (e.g., “She forgot what her mom told her to buy”) over the incorrect interpretations of the metaphors (e.g., “She bought candies with the money”). Thus, in contrast to previous metaphor studies that tested children’s abilities to discriminate between different kinds of metaphors (e.g., Gentner & Clement, 1988; Silberstein et al., 1982), the more recent metaphor studies (Özçalişkan, 2005, 2007; Pouscoulous & Tomasello, 2020) tested children’s ability to discriminate between metaphors versus incorrect or nonsensical statements or interpretations.

The latter studies suggest that even young children may understand metaphors before they can produce them—and thus that the basic cognitive capacities underlying metaphorical competence may be in place earlier than previously believed. Notably, however, these studies strictly investigated preschoolers’ capacity to understand metaphors based on shared concrete surface-level features (e.g., color) and space-motion metaphors, which are heavily conventionalized and may be based on innate bodily mapping or cross-modal perception (Casasanto, 2017; Lakoff & Johnson, 1980; Lourenco & Longo, 2011; Pitt & Casasanto, 2020). Thus, it is still largely unknown whether preschoolers’ capacity to understand metaphors extends to metaphors based on more abstract similarities. Notably, it is this more abstract kind of metaphor that is hypothesized to be particularly useful for reasoning and learning (Zhu et al., 2024). Metaphors allow for the reformulation of complex ideas in terms of simpler ones (e.g., “love is a journey”; “conscience is a man’s compass”; Thibodeau et al., 2017). Given the diverse benefits that abstract metaphor comprehension may hold for learning and education more generally, it would be not only scientifically but also practically significant to show that preschoolers’ capacity to understand and use metaphors extends beyond perceptual metaphors and heavily conventionalized, conceptually “close” motion-space metaphors (Lakoff & Johnson, 1980).

One recent study showed that preschoolers can learn from abstract metaphors (Zhu & Gopnik, 2023). In this experimental paradigm, preschoolers heard information about novel toys conveyed through metaphors that described the objects’ functional capacities. For example, “Daxes are suns” conveys that the novel toys, daxes, have the capacity to light up. Preschoolers successfully formed metaphor-consistent inferences about the functions of the novel toys from these functional metaphors. These findings suggest that children understand abstract metaphors about artifact functions, since they are able to learn from them. While these findings provide promising initial evidence of preschoolers’ metaphor comprehension, additional converging evidence is required to demonstrate that preschoolers indeed possess a robust understanding of metaphors, which can be further leveraged to support a complex suite of cognitive processes.

The present research aims to do exactly this. Given the recent findings suggesting that young children’s aptitude for relational reasoning and metaphor comprehension may be stronger than previously believed, and given the recent finding that children can learn about artifact functions from metaphors, the present study further investigates whether young children might be able to understand abstract metaphors at an earlier developmental timepoint than researchers initially estimated. Here, we will use the term *functional metaphor* to refer to metaphors that draw equivalences based on abstract, relational, or structural commonalities (including but not limited to the designed functions of artifacts). The conceptual foils to functional metaphors are *perceptual metaphors*, which draw equivalences based on surface-level, featural, or semantic-associative commonalities. In the present experiments, however, we pit functional metaphors against *nonsense statements* to directly probe children’s appreciation (or lack thereof) of metaphor meanings based on abstract similarities.

In this present research, we conducted multiple exploratory experiments demonstrating preschoolers’ competence with functional metaphors. Like the earlier experiments that found evidence for early comprehension of perceptual metaphors, the current experiments use a forced-choice technique to minimize cognitive demands. In particular, following recent approaches, Experiments 1 and 3 test children’s ability to discriminate between functional metaphors and nonsense statements. We also ask children to provide explicit justifications for their judgments, potentially providing even more convincing evidence that they interpret functional metaphors correctly. Moreover, we compare their performance on metaphors versus similes. Given that causal frameworks – which require learners to relate an initial state to an end state via some causal process – can induce a relational mindset (Goddu et al., 2020; Walker et al., 2016; Walker & Gopnik, 2017), we also explored whether causal framing might facilitate preschoolers’ metaphor comprehension.

Experiment 1A showed that preschoolers rated functional metaphors (e.g., “roofs are hats”) as significantly “smarter” than nonsense statements (e.g., “boats are skirts”) in a metalinguistic judgment paradigm. Experiment 1B validated this novel paradigm with adults. Experiment 2 showed that preschoolers preferred functional explanations (e.g., “roofs and hats both keep you dry”) over perceptual explanations (e.g., “roofs and hats both have pointy tops”) when interpreting the functional metaphors used in Experiment 1A, thus demonstrating that preschoolers interpreted functional metaphors in an adult-like fashion. Finally, Experiment 3 showed that preschoolers preferred functional metaphors (e.g., “roofs are hats”) over nonsense statements (e.g., “roofs are scissors”) in a dichotomous-choice paradigm, providing the most robust evidence that children understand functional metaphors. Taken together, these results suggest that preschoolers understand complex metaphors based on abstract similarities, such as shared function.

## EXPERIMENT 1A

In Experiment 1A, we use a novel metalinguistic judgment paradigm to explore preschoolers’ metaphor comprehension abilities. In particular, we asked children to judge whether functional metaphors and nonsense statements were “silly” or “smart”. Though researchers have previously suggested that preschoolers might struggle to form metalinguistic judgments about metaphors (Pouscoulous & Tomasello, 2020), our novel dichotomous-choice paradigm is relatively simple. If children succeed in this task, they demonstrate that they can both understand and evaluate functional metaphors. Functional metaphors clearly don’t make sense if they are taken literally (e.g., roofs are not comparable to hats). Thus, from a literal perspective, it is possible that children will judge functional metaphors in the same way as nonsense statements. On the other hand, if these utterances are treated as metaphors, functional metaphors might not only make sense but might also be illuminating. Discriminating between metaphors and nonsense statements at all, then, already suggests some understanding of metaphors. But judging that metaphors are smart and nonsense statements are silly would show that children understand metaphors in a more adult-like way.

This experiment also tested multiple reasons why preschoolers might struggle with metaphor comprehension. While one possibility is that preschoolers struggle with relational reasoning, another possibility is that preschoolers struggle with non-literal interpretations (Allen & Butler, 2020; Reynolds & Ortony, 1980; Vivaldi & Allen, 2021). Consequently, Experiment 1A involves conditions with both metaphors, which are non-literal (e.g., “Clouds are sponges”), and similes, which are literal (e.g., “Clouds are *like* sponges”). Preschoolers’ ability to understand similes would indicate an early competency with relational reasoning—the mapping of abstract similarities between entities—whereas preschoolers’ ability to understand metaphors would indicate early competencies with both relational reasoning and non-literal language. While relational reasoning underlies both metaphor and simile comprehension, the two phenomena are also distinct: adults seem to favor metaphors over similes, reporting that metaphors are more interesting (Roberts & Kreuz, 1994) and cognitively “forceful” than similes (Glucksberg & Keysar, 1990).

Finally, in light of recent work that has suggested that causal framing may have a facilitative effect on relational reasoning (Goddu et al., 2020; Walker et al., 2016; Walker & Gopnik, 2017), we also included causal and non-causal training trials to test for facilitative effects of causal framing on metaphor comprehension.

### Methods

Children in the causal framing condition received a warm-up task involving the causal transformation of objects on a conveyor belt, whereas children in the control conditions received a similar non-causal warm-up task or no warm-up task. Then, all children were given a novel metaphor comprehension task, in which they must make “smart” or “silly” metalinguistic judgments of functional metaphors and nonsense statements. Moreover, given that some previous research suggests that children understand similes more easily than metaphors (Reynolds & Ortony, 1980; but see also Winner et al., 1980), we ran a causal condition with similes (e.g., “Roofs are *like* hats”) as well as a causal condition with metaphors (e.g., “Roofs are hats”). Some of the metaphors in the experiment were taken from previous studies (e.g., “Moons are lightbulbs”; Gentner, 1988), while others (e.g., “Pools are bowls”) were newly-generated.

#### Participants.

We tested 32 children per condition, leading to a total of 128 4- to 5-year-olds who participated in the study (*M* = 4.86 years; *SD* = .51 years; range = 4.01–5.88 years; 61 males, 67 females). Researchers tested an additional two children, whose data were excluded due to failure to complete the study (one child) and external interference (one child). Children were recruited and tested in a quiet preschool or museum setting. All experiments in this paper lasted approximately five to ten minutes, and were conducted independently (i.e., participants did not complete additional studies during the same testing session). All sample sizes in this paper, though not formally preregistered, were set prior to testing based on counterbalancing requirements. Since our recruitment techniques drew from local convenience samples, participants were predominantly White and upper middle class across all experiments. All experiments in this paper were approved by the university’s Committee for the Protection of Human Subjects. All parents of child participants provided informed consent.

#### Stimuli and Procedure.

The experimenter presented participants with the stimuli on a laptop computer. Each child participated in one of four conditions. The *Causal Metaphor* condition involved causal training trials prior to the test trials, and used metaphors throughout. The *Causal Simile* condition involved causal training trials prior to the test trials, and used similes throughout. The *Control Simile* condition involved non-causal training trials prior to the test trials, and used similes throughout. Finally, the *Baseline Simile* condition involved only test trials using similes. During the test trials, all participants were presented with metaphors and nonsense statements, and had to differentiate between the two kinds of utterances. For the full list of metaphors, similes, and nonsense statements used in each experiment, see Supplementary Materials on OSF (https://osf.io/cpk92/).

#### Causal Metaphor Training Trials.

In the Causal Metaphor training trials, participants saw the components of the metaphor in a causal context, specifically as objects undergoing causal transformations. These trials were modelled after another experiment (Goddu et al., 2020) that demonstrated preschoolers’ understanding of abstract relations in the context of causal transformations. For example, children who saw a wizard turn a small apple into a large apple predicted that the wizard’s action on a new object would lead to a transformation that exemplified the same relation (e.g., turn a small dog into a large dog).

In the Causal Metaphor training trials, the experimenter introduced the task by saying, “Hi! I’m going to tell you about a person named Annie! Annie works in a factory with a super cool purple machine. Let’s watch Annie use the purple machine and see what happens.” Each training trial presented participants with two metaphors. During the first part of the training trial, participants saw an object (e.g., a bird) on the left side of a purple conveyor belt. The experimenter pointed and named the object (e.g., “Look! Annie has a bird!”) The object traveled down the conveyor belt, and in the middle of the conveyor belt, a purple box came down and covered the object. When the purple box went up again, it revealed another object (e.g., a hot air balloon). The second object then traveled to the right side of the conveyor belt. Finally, the experimenter used the two objects from the conveyor belt in a metaphoric utterance (e.g., “Annie says, ‘Birds are hot air balloons!’”)

During the second part of the training trial, participants saw a new object (e.g., a sleeping bag) on the right side of the conveyor belt. Two objects appeared below the conveyor belt: one that was a *functional match*, namely an object that shared the same function (e.g., a glove), and one that was an *object match*, namely an object from the previous trial (e.g., a hot air balloon). The experimenter pointed to and named the object, and then prompted participants to find a match for the object on the conveyor belt (e.g., “Look! Annie has a sleeping bag! This time, Annie is going to use the machine on the sleeping bag. Do you think the sleeping bag is going to turn into a glove or a hot air balloon?”) After the participant made a prediction by selecting one of the objects below, the participant received feedback: the new object (e.g., the sleeping bag) went down the conveyor belt, which always causally transformed the object into its function-matched counterpart (i.e., a glove), regardless of what object the participant chose. To end the trial, the experimenter used the two objects from the conveyor belt in a metaphoric utterance (e.g., “Annie says, ‘Sleeping bags are gloves!’”).

Each participant received four training trials with a total of eight metaphors. Each trial’s structure followed the design described above, in which the participant watched an object go down the conveyor belt, and then was asked to predict what the novel object on the conveyor belt will turn into. Participants received feedback on each of their choices. The order of the four training trials was randomized and the left-right placement of the function match and the object match was counterbalanced across participants. The experimenter pointed to the objects on the screen (e.g., bird, hot air balloon, glove, sleeping bag) as she named them.

It is worth noting that though the statements in the Causal Metaphor condition follow a standard “X is Y” metaphor form, these statements might not be considered metaphors because the statements are literally true: one object in the statement undergoes a causal transformation and literally turns into the other object. However, whether or not preschoolers believe these statements to be literal or non-literal should not affect whether they select the functional match or the object match.

#### Causal Simile Training Trials.

The Causal Simile training trials were identical to the Causal Metaphor training trials, except all utterances were similes (e.g., “Annie says, ‘Birds are *like* hot air balloons’”) rather than metaphors. Given that some previous work suggests that young children may have difficulty with non-literal language (Reynolds & Ortony, 1980), we ran the Causal Simile condition as well as the Causal Metaphor condition to see whether literal, as opposed to non-literal, statements might increase the accuracy of participants’ responses.

#### Control Simile Training Trials.

The Control Simile training trials were identical to the Causal Simile training trials, except that the objects were not presented in a causal context. Thus, there was no conveyor belt. Rather, Annie simply uttered statements about objects that appeared on the screen, providing participants with the same statements about objects, but without causal framing. During the second part of the training trial, when prompting participants to match the initial object with either a function match or an object match, the experimenter asked what the object was more similar to rather than what the object would turn into (e.g., “Do you think the sleeping bag *is like* a glove or a hot air balloon?”), since the objects did not causally transform into one another. The experimenter still gave participants feedback on their responses.

#### Baseline Simile Condition.

In the Baseline Simile condition, participants were not presented with training trials. Instead, participants in this condition participated in the test trials without any previous training.

#### Test Trials.

During the Test Trials, we deliberately emphasized to participants that they were playing a new game with a new character, so that the test trial metaphors – which involve objects merely appearing onscreen, rather than undergoing causal transformations – are more likely to be interpreted as non-literal statements. The experimenter introduced the test trials by saying, “Now let’s play a new game. In this game, we’re going to play with Annie’s friend Meg. Meg is going to say things and we need your help figuring out whether what Meg said is smart or silly!” The experimenter pointed at a green happy face on the computer screen while saying “smart” and a red sad face on the computer screen while saying “silly”. Then, the experimenter showed Meg with two objects (e.g., a roof and a hat) and said, “Meg says, ‘Roofs are hats!’ Is what Meg said smart or silly?”. The experimenter pointed to the objects on the screen as she named them, and to the happy face and the sad face while saying “smart” and “silly” respectively. Once the participant answered by providing a verbal response (e.g., “I think it’s smart”) or pointing at the happy or sad face, the experimenter began the next trial. No feedback was provided.

The last trial was always a metaphor. On the last trial, after participants had provided a smart/silly response, the experimenter asked for an open-ended explanation about the similarity between the two components of the metaphor (e.g., “How are windows like eyes?”).

There were sixteen test trials total: eight metaphors (e.g., “Clouds are sponges”; “Tires are shoes”) and eight nonsense statements (e.g., “Dogs are scissors”; “Pennies are sunglasses”). We counterbalanced whether participants received a metaphor or nonsense statement first. In order to minimize executive function demands that could influence metaphor comprehension (Carriedo et al., 2016), the “smart” option (happy face) was always on the right and the “silly” option (sad face) was always on the left. No more than three of the same kind of trial appeared consecutively, and the last trial was always a metaphor. Each of the eight metaphors appeared as the last trial an equal number of times (e.g., within each condition, children were asked to explain how clouds are like sponges as frequently as they were asked to explain how tires are like shoes).

In the Causal Metaphor condition, all statements were presented non-literally (e.g., “Clouds are sponges”) whereas in the Causal Simile, Control Simile, and Baseline Simile conditions, all statements were presented literally (e.g., “Clouds are *like* sponges”).

### Results & Discussion

#### Training Trials.

First, we examined whether presenting objects in a causal context changed children’s likelihood of selecting the functional match or the object match during the training trials. A between-subjects ANOVA with Condition (Causal Metaphor, Causal Simile, Control Simile) as the independent variable and Response (Functional Match, Object Match) as the dependent variable yielded a main effect of Condition, *F*(2, 93) = 12.72, *p* < .001. Specifically, children in the Causal Metaphor condition selected the functional match significantly more frequently than children in the Control Simile condition, *t*(62) = 4.28, *p* < .001. Children in the Causal Simile condition also selected the functional match significantly more frequently than children in the Control Simile condition, *t*(62) = 4.19, *p* < .001. There was no difference in children’s performance between the Causal Metaphor and Causal Simile conditions, *t*(62) = .14, *p* = .89. Thus, we found that children in the two causal conditions selected the functional match more frequently than in the control condition.

Additionally, we examined whether children were significantly above chance at selecting the functional match over the object match in each condition. Since there were three experimental groups being compared to chance, we used a Bonferroni correction for multiple comparisons, leading to an adjusted alpha of .017. (We analyzed all results with multiple comparisons in this paper using Bonferroni corrections, but only report adjusted alphas when they impact interpretations of significance or non-significance in the results.) We found that children selected the functional match at above chance levels in the Causal Metaphor condition, *M* = 85.94%, *SE* = 3.71%, *t*(31) = 9.68, *p* < .001, and the Causal Simile condition, *M* = 86.72%, *SE* = 4.20%, *t*(31) = 8.75, *p* = .001. However, children were at chance selecting between the functional match and the object match in the Control Simile condition, *M* = 60.16%, *SE* = 4.74%, *t*(31) = 2.14, *p* = .04.

#### Test Trials.

In order to determine whether children were able to differentiate between metaphors and nonsense statements, we created a Composite Score (percentage of metaphors rated as “smart” subtracted by percentage of nonsense statements rated as “smart”) for each child. A child who rated all metaphors as “smart” and all nonsense statements as “silly” would have a score of 1, whereas a child who rated all metaphors *and* nonsense statements as “smart” would have a score of 0. Thus, the Composite Score assessed children’s performance on both metaphor and nonsense statement trials. We opted to use a Composite Score involving raw differences as opposed to a more standard z-transformed discriminability metric (e.g., d-prime analyses; Stanislaw & Todorov, 1999) because a metric involving raw data was more consistent with the rest of the analyses, which also involve raw scores (e.g., overall accuracy scores).

In order to investigate whether causal framing would facilitate performance on the metaphor task, we ran a between-subjects ANOVA with Condition (Causal Metaphor, Causal Simile, Control Simile, Baseline Simile) as the independent variable and Composite Score as the dependent variable. There was no effect of Condition on Composite Score, *F*(3, 125) = .30, *p* = .82. Similarly, a linear regression comparing Composite Scores in the three training conditions to the baseline condition showed no significant difference between the Baseline Simile condition, *M* = 10.94%, *SE* = 7.96%, and any of the other conditions, including the Causal Metaphor condition, *M* = 15.63%, *SE* = 5.38%, *β* = .14, *p* = .57, Causal Simile condition, *M* = 17.19%, *SE* = 5.32%, *β* = .19, *p* = .45, and Control Simile condition, *M* = 10.94%, *SE* = 4.75%, *β* < .001, *p* = 1.00.

Since we did not find a significant difference between any of the conditions, we aggregated data across conditions and analyzed them together. From the aggregated Composite Scores, we find that children performed significantly above chance on the test trials, *M* = 13.67%, *SE* = 2.91%, *t*(127) = 4.70, *p* < .001, *d* = .42. However, while children rated nonsense statements as “silly” significantly more frequently than chance, *M* = 59.28%, *SE* = 2.78%, *t*(127) = 3.34, *p* < .001, they did not rate metaphors as “smart” significantly more frequently than chance, *M* = 54.39%, *SE* = 2.43%, *t*(127) = 1.81, *p* = .07.

#### Explanations.

We examined the explanations that children gave for how the two components of a metaphor were alike (e.g., “How is a tire like a shoe?”). There were 128 explanations total, as each child provided an explanation on the final trial. Explanations were coded blind to participants’ responses in the training and test trials. Explanations fell into three categories: irrelevant, perceptual, and functional. Irrelevant explanations were non-responses (e.g., “I don’t know”) or irrelevant (e.g., “I have a tire swing”) and comprised 49% of all explanations. Perceptual explanations accurately referred to perceptual similarities between the target objects (e.g., “because a cloud is fluffy and a sponge is fluffy” when explaining the metaphor “clouds are sponges”; “because they’re both flat” when explaining the metaphor “grasses are rugs”) comprised 25% of all explanations. Functional explanations accurately referred to functional similarities between the target objects (e.g., “because you can see through a window and that’s why they’re like eyes” when explaining the metaphor “eyes are windows”; “because they both protect your head” when explaining the metaphor “roofs are hats”) and comprised 26% of all explanations. Two coders coded all explanations. Intercoder reliability was 95%, converging on the same category for 122 out of 128 explanations. The categorization of the remaining 6 explanations was resolved through discussion.

**Figure 1.**
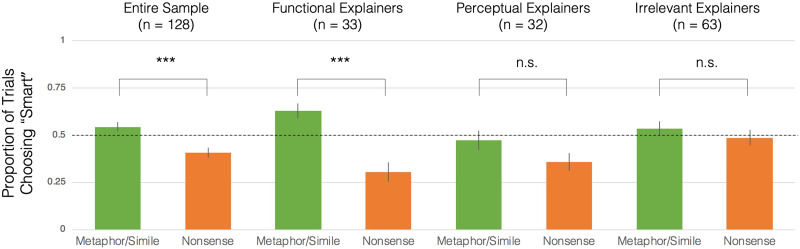
Test trial data from preschoolers. Error bars show 1 standard error from subject-level data.

We analyzed data from the children who provided functional explanations, perceptual explanations, and irrelevant explanations separately, examining whether the composite scores, metaphor ratings, and nonsense ratings were significant for each group of explanations (see Figure 1). Since there were a total of nine comparisons against chance, we used a Bonferroni correction for multiple comparisons, leading to an adjusted alpha of .006. We find that the children who provided functional explanations (*n* = 33) were able to distinguish between metaphors and nonsense statements: the functional explainers had Composite Score above chance levels, *M* = 32.58%, *SE* = 5.24%, *t*(32) = 6.21, *p* < .001, and were significantly likely to rate metaphors as “smart”, *M* = 62.88%, *SE* = 3.79%, *t*(32) = 3.40, *p* = .002 and nonsense statements as “silly”, *M* = 69.70%, *SE* = 5.13%, *t*(32) = 3.84, *p* < .001. In contrast, the perceptual explainers (*n* = 32) had an average Composite Score that was not significantly different from chance levels, *M* = 11.33%, *SE* = 6.11%, *t*(31) = 1.86, *p* = .07, and performed at chance on ratings for both metaphors, *M* = 47.27%, *SE* = 5.04%, *t*(31) = .54, *p* = .59, and nonsense statements, *M* = 64.07%, *SE* = 4.72%, *t*(31) = 2.98, *p* = .006. The irrelevant explainers (n = 63) also had an average Composite Score that was not significantly different from chance levels, *M* = 4.96%, *SE* = 3.74%, *t*(62) = 1.33, *p* = .19, and performed at chance on ratings of both metaphors, *M* = 53.57%, *SE* = 3.64%, *t*(62) = .98, *p* = .33, and nonsense statements, *M* = 51.39%, *SE* = 4.15%, *t*(62) = .33, *p* = .74. Thus, the subset of children who provided explanations involving functional similarity performed above chance on all measures of metaphor comprehension, and their performance drove the success of the entire sample.

Additionally, we found no age differences between the three groups of explainers. A one-way between-subjects ANOVA found no effect between explanation type (Functional, Perceptual, Irrelevant) and age, *F*(2, 125) = 2.17, *p* = .12. Similarly, using Welch’s *t*-test to account for unequal variance due to the different sample sizes of the explanation groups, we find no difference in the ages of children across different explanation groups. Specifically, we find that the age of children who provided functional explanations (*M* = 5.01 years, *SE* = .09, range = 4.17–5.88 years) was not significantly different from the age of children who provided perceptual explanations (*M* = 4.85 years, *SE* = .09, range = 4.01–5.87 years), *t*(62.88) = 1.22, *p* = .23, or the age of children who provided irrelevant explanations (*M* = 4.78 years, *SE* = .06, range = 4.02–5.88 years) after a Bonferroni correction for multiple comparisons, *t*(64.72) = 2.09, *p* = .04. Likewise, the age of children who provided perceptual explanations was also not signficantly different from the age of children who provided irrelevant explanations, *t*(61.42) = .65, *p* = .52. We also conducted Welch’s *t*-tests to examine whether the ages of any of the explanation groups was significantly different from the overall age of the entire sample of 128 children. We found no significant difference between the age of the children in the entire sample and the age of the children who provided functional explanations, *t*(50.13) = 1.52, *p* = .14, the age of the children who provided functional explanations, *t*(47.60) = 0.05, *p* = .96, and the age of the children who provided irrelevant explanations, *t*(125.17) = .98, *p* = .33. Thus, all our age analyses showed no age differences across the three different types of explainers.

Finally, we also investigated whether explanation type varied across the four training conditions. We found no relation between condition (Causal Metaphor, Causal Simile, Control Simile, Baseline Simile) and explanation type (Functional, Perceptual, Irrelevant), *χ*^2^ (6, *N* = 128) = 9.39, *p* = 0.15.

#### Alternative Analyses.

Consistent with previous metaphor comprehension studies (Özçalişkan, 2007; Pouscoulous & Tomasello, 2020), we chose to conduct linear analyses (e.g., *t*-tests) on subject-level data. However, given that all experimental paradigms in this paper use a dichotomous forced-choice paradigm, it was also useful to conduct alternative analyses on trial-level data. Specifically, in addition to the subject-level frequentist analyses reported in this paper, we also conducted all analyses using trial-level Bayesian analyses. All Bayesian analyses conducted in this series of experiments were exploratory analyses. We analyzed the data with the *brms* R package (Bürkner, 2016) using the default flat priors. Each model underwent a warm-up period of 1000 iterations, followed by four sampling chains with 2000 iterations each. All results converged with subject-level frequentist analyses, except for four results in Experiment 1A and one result in Experiment 3 (detailed in Experiment 3’s Results section). In contrast to subject-level frequentist analyses, a trial-level Bayesian logistic regression examining response (i.e., “smart” or “silly”) by statement type (i.e., metaphor or nonsense statement) with subject as a random intercept showed that preschoolers rated nonsense statements as silly, posterior mean accuracy = 59.78%, 95% credible interval [55.86%, 63.42%], probability that the estimate is above chance levels (log odds > 0) = 1.00, *and* rated functional metaphors as smart, posterior mean accuracy = 54.66%, 95% credible interval [50.96%, 58.56%], probability that the estimate is above chance levels (log odds > 0) = .99. Moreover, in contrast to subject-level frequentist analyses, a trial-level Bayesian logistic regression examining training trial accuracy (i.e., selecting the functional match) by condition (i.e., Causal Metaphor, Causal Simile, and Control Simile) with subject as a random intercept suggested that even preschoolers in the Control Simile condition selected the functional match over the perceptual match, posterior mean accuracy = 62.21%, 95% credible interval [49.28%, 74.65%], probability that the estimate is above chance levels (log odds > 0) = .97.

Similarly, we found some differences in overall task performance using these two kinds of analyses. To measure overall task performance in the subject-level frequentist analyses, we used a composite score – that is, a difference measure reflecting participants’ ability to differentiate between metaphors and nonsense statements (i.e., the percentage of metaphors rated as “smart” subtracted by percentage of nonsense statements rated as “smart”). While composite scores are a more sensitive measure of task performance than overall task accuracy, calculating composite scores requires subject-level rather than trial-level analyses, because composite scores account for correct responses *and* penalize incorrect responses, over multiple trials. Consequently, to measure overall task performance in the trial-level Bayesian analyses, we instead used overall task accuracy – that is, an absolute measure of the total percentage of correct responses (i.e., the percentage of metaphors rated as “smart” and the percentage of nonsense statements rated as “silly”). The overall task accuracy measure only accounted for correct responses, and did not penalize for incorrect responses. Using overall task accuracy, rather than composite scores, allowed for trial-level analyses, such that participants’ responses on individual trials could be coded as separate data points in the analyses.

A trial-level Bayesian logistic regression examining overall task accuracy by explanation type (i.e., functional, perceptual, or irrelevant) with subject as a random intercept showed that functional explainers differentiated between functional metaphors and nonsense statements, posterior mean accuracy = 66.89%, 95% credible interval [61.08%, 71.97%], probability that the estimate is above chance levels (log odds > 0) = 1.00, *and* perceptual explainers differentiated between functional metaphors and nonsense statements, posterior mean accuracy = 55.95%, 95% credible interval [50.25%, 61.70%], probability that the estimate is above chance levels (log odds > 0) = .98. Irrelevant explainers did not differentiate between functional metaphors and nonsense statements, posterior mean accuracy = 52.55%, 95% credible interval [48.73%, 57.01%], probability that the estimate is above chance levels (log odds > 0) = .89.

In particular, a trial-level Bayesian logistic regression examining overall accuracy (i.e., rating functional metaphors as “smart” and nonsense statements as “silly”) by explanation type (i.e., functional, perceptual, or irrelevant) and statement type (i.e., metaphor or nonsense statement) with subject as a random intercept showed that functional explainers rated metaphors as “smart”, posterior mean accuracy = 63.52%, 95% credible interval [56.66%, 70.20%], probability that the estimate is above chance levels (log odds > 0) = 1.00, and nonsense statements as “silly”, posterior mean accuracy = 70.55%, 95% credible interval [64.14%, 76.84%], probability that the estimate is above chance levels (log odds > 0) = 1.00. In contrast, perceptual explainers did not rate metaphors as “smart”, posterior mean accuracy = 47.19%, 95% credible interval [40.10%, 54.17%], probability that the estimate is above chance levels (log odds > 0) = .22, but did rate nonsense statements as “silly”, posterior mean accuracy = 64.47%, 95% credible interval [57.79%, 71.44%], probability that the estimate is above chance levels (log odds > 0) = 1.00. Irrelevant explainers did not rate metaphors as “smart”, posterior mean accuracy = 53.75%, 95% credible interval [48.44%, 58.75%], probability that the estimate is above chance levels (log odds > 0) = .92, or nonsense statements as “silly”, posterior mean accuracy = 51.39%, 95% credible interval [46.22%, 56.61%], probability that the estimate is above chance levels (log odds > 0) = .69. Thus, while subject-level frequentist analyses suggest that only functional explainers succeed at the present task, trial-level Bayesian analyses suggest that perceptual explainers also succeeded at the task. In particular, Bayesian analyses suggest that functional explainers rated functional metaphors as “smart” and nonsense statements as “silly”, while perceptual explainers only rated nonsense statements as “silly”. For a full comparison of subject-level frequentist analyses and Bayesian analyses, including converging results, see “Bayesian Analyses” on OSF (https://osf.io/cpk92/).

Overall, our novel metalinguistic judgment paradigm showed that preschoolers already possess some competence with metaphor comprehension and relational reasoning: as a group, preschoolers distinguished between functional metaphors and nonsense statements. This effect was primarily driven by the quarter of the children who explicitly noted the functional similarities between objects in their explanations. Additionally, we found no difference in children’s performance on similes and metaphors, suggesting that preschoolers understand literal and non-literal language equally well.

Consistent with previous research (Goddu et al., 2020; Walker et al., 2016; Walker & Gopnik, 2017), we find that introducing a causal framework encouraged preschoolers to adopt a relational mindset, such that they selected the functional matches over the object matches during the causal training trials. However, there was no effect of the causal framework training trials on the metaphor comprehension test trials.

## EXPERIMENT 1B

Since our “smart” and “silly” judgment task is a novel experimental paradigm, we ran a sample of adults in order to validate the paradigm. Comprehension of novel metaphors can be challenging for adults (Blasko & Connine, 1993; Bowdle & Gentner, 2005) as well as children (Demorest et al., 1983; Silberstein et al., 1982; Winner et al., 1980). Our novel paradigm may be somewhat pragmatically odd, as it may be unclear what it means for an utterance to be “smart” or “silly”. Thus, we wished to demonstrate that adults could distinguish between metaphors and nonsense statements and would rate metaphors as “smart” and nonsense statements as “silly”.

### Methods

#### Participants.

We tested 32 participants per condition, resulting in a total of 64 adult participants (*M* = 24.70 years; *SD* = 5.97 years; range = 18.62–41.02 years; 25 males, 39 females). Researchers tested an additional three participants, whose data were excluded due to experimenter error (two participants) and external interference (one participant). Adults were recruited and tested in a university lab or other quiet on-campus setting. All participants provided informed consent.

#### Stimuli and Procedure.

We ran adults on either the Causal Metaphor condition or Causal Simile condition. The stimuli and procedure of these two conditions are identical to those detailed in Experiment 1A.

### Results & Discussion

#### Training Trials.

There was no significant difference in training trial performance between conditions; indeed, adults performed identically in the two conditions, *t*(62) = 0, *p* = 1.00. Adults were almost at ceiling in both conditions. Participants were significantly more likely to pick the functional match than the object match in the Causal Metaphor condition, *M* = 93.75%, *SE* = 1.94%, *t*(31) = 22.50, *p* < .001, and the Causal Simile condition, *M* = 93.75%, *SE* = 1.94%, *t*(31) = 22.50, *p* < .001.

#### Test Trials.

We again created Composite Scores (percentage of metaphors rated as “smart” subtracted by percentage of nonsense statements rated as “smart”) for each participant. Aggregating together adults’ responses from both the Causal Metaphor and Causal Simile conditions, we found that overall Accuracy, as measured by the Composite Scores, is significantly different from chance levels, *t*(63) = 24.50, *p* < .001, *d* = 3.06. Additionally, we found that Accuracy is significantly different between the conditions, *t*(62) = 2.24, *p* = .03, with Accuracy being greater in the Causal Simile condition, *M* = 86.33%, *SE* = 2.94%, than in the Causal Metaphor condition, *M* = 72.27%, *SE* = 5.55%. Regardless, adults were able to distinguish between metaphors and nonsense statements at above-chance levels in both the Causal Metaphor condition, *t*(31) = 13.02, *p* < .001, and Causal Simile condition, *t*(31) = 29.41, *p* < .001. The difference in Accuracy across conditions was driven by differences in responses to metaphors. While there was no significant difference between adults’ ratings of the nonsense statements between the Causal Metaphor and Causal Simile conditions, *t*(62) = .36, *p* = .72, adults in the Causal Metaphor condition rated the metaphors as “smart” significantly less frequently than adults in the Causal Simile condition, *t*(62) = 2.21, *p* = .03. 4 out of 32 adults in the Causal Metaphor condition rated all metaphor and nonsense statements as “silly”, thus driving down the overall percentage of metaphors rated as “smart” in the Causal Metaphor condition. In contrast, none of the adults in the Causal Simile condition rated all metaphors and nonsense statements as “silly”.

Despite differences in the metaphor ratings between the Causal Metaphor and Causal Simile conditions, we found that adults in both conditions were above chance at rating both metaphors and nonsense statements. In the Causal Metaphor condition, adults were significantly above chance at rating the metaphors as “smart”, *M* = 79.30%, *SE* = 5.66%, *t* (31) = 5.18, *p* < .001, and the nonsense statements as “silly”, *M* = 92.97%, *SE* = 2.24%, *t*(31) = 19.18, *p* < .001. Similarly, in the Causal Simile condition, adults were significantly above chance at rating the metaphors as “smart”, *M* = 92.19%, *SE* = 1.46%, *t*(31) = 28.93, *p* < .001, and the nonsense statements as “silly”, *M* = 94.14%, *SE* = 2.38%, *t*(31) = 18.55, *p* < .001. Moreover, 78% of adults in the Causal Metaphor condition and 97% of adults in the Causal Simile condition provided explanations based on functional similarity on the last trial.

The results of Experiment 1B validate our paradigm, by showing that adults in both conditions judge metaphors as significantly “smart” and nonsense statements as significantly “silly.” However, consistent with previous work demonstrating that novel metaphor comprehension is difficult even for adults (Blasko & Connine, 1993), we find that adults are not always at ceiling at this task, especially in terms of rating metaphors as “smart”. Interestingly, while there was no difference between preschoolers’ “smartness” ratings of metaphors and similes, adults rated similes as smarter than metaphors. This result is consistent with previous work showing that adults prefer novel comparisons, such as the stimuli used in this experiment, in literal simile form rather than non-literal metaphor form (Bowdle & Gentner, 2005).

## EXPERIMENT 2

The results of Experiment 1A and 1B demonstrate that both preschoolers and adults are capable of differentiating metaphors from nonsense statements. However, an outstanding question is whether preschoolers’ performance in Experiment 1A was actually driven by their understanding of functional similarities between objects in the functional metaphors. Although a quarter of preschoolers provided functional explanations to justify their choices in Experiment 1A, it is still possible that preschoolers do not have sufficient understanding of the objects’ functions (e.g., preschoolers do not possess the background knowledge that clouds store water), or judge the metaphors based on other kinds of non-functional similarities (e.g., preschoolers think clouds and sponges are alike because both are fluffy, not because both hold water). Using an established paradigm (Gentner, 1988), Experiment 2 seeks to validate and strengthen the results of Experiment 1A, by demonstrating that preschoolers notice the functional similarities in the functional metaphors used in Experiment 1A. Thus, Experiment 2 explores whether preschoolers preferred *functional explanations* (i.e., explanations involving functional similarities between two concepts) over *perceptual explanations* (i.e., explanations involving perceptual similarities between two concepts) when interpreting functional metaphors.

### Methods

#### Participants.

We tested 24 participants per condition in two conditions, leading to a total of 48 children who participated in the study (*M* = 5.02 years; *SD* = .62 years; range = 4.01–5.93 years; 28 males, 20 females). Researchers tested an additional participant, whose data were excluded because they failed the attention check. Children were recruited and tested in a quiet preschool or museum setting.

#### Stimuli and Procedure.

As in Experiment 1A, the experimenter presented participants with the stimuli on a laptop computer. Each child was presented with four training trials and eight test trials, and participated in one of two conditions: the Causal Metaphor condition or the Control Metaphor condition. The training trials differed between the two conditions, but the test trials were identical between the two conditions. Experiment 2 used the same functional metaphors as Experiment 1, in both the training and test phase.

#### Training Trials.

The Causal Metaphor training trials in Experiment 2 were identical to the Causal Metaphor training trials in Experiment 1A. The Control Metaphor training trials in Experiment 2 were almost identical to the Control Simile training trials in Experiment 1, except that all statements were presented as metaphors instead of similes.

#### Test Trials.

Participants in both the Causal Metaphor condition and Control Metaphor condition received identical test trials. The experimenter introduced the test trials by saying, “You did such a good job at that game! Now we’re going to play a new game! In this game we’re going to play with Annie’s friend Meg. Meg is going to ask questions. One person will give her an answer to her question. Then, another person will give her a different answer to her question. Your job is to point at the person who gives Meg the better answer. Let’s play!”

On each trial, Meg posed a question (e.g., “How are clouds sponges?”) as the two objects in the metaphor (e.g., a cloud and a sponge) appeared on the screen. Two people then appeared at the bottom of the screen. First, one person appeared on the left and provided an explanation (e.g., “Clouds are sponges because both give water!”). Then, another person appeared on the right and provide an explanation (e.g., “Clouds are sponges because both are fluffy!”). The experimenter prompted the participant to choose an explanation by asking, “Whose answer is better?” Once the participant answered by pointing at one of the two people or providing a verbal response (e.g., “The one who said fluffy”), the experimenter began the next trial. No feedback was provided.

There were eight test trials total, with each trial involving one of the eight functional metaphors from the test trials in Experiment 1. We counterbalanced whether the functional explanation appeared on the left or the right.

Since we did not ask participants to provide their own explanations in Experiment 2, we added an attention check at the end of the study. In the attention check trial, Meg asked, “What is this animal called?” while a picture of a dog appeared on the screen. The person on the left provided the correct description (i.e., “The animal is a dog!”) and the person on the right provided an incorrect description (i.e., “The animal is a fish!”). Children needed to select the correct description in order to pass the attention check.

### Results & Discussion

#### Training Trials.

There was no significant difference in training trial performance between the Causal and Control conditions, *t*(46) = .99, *p* = .33. In fact, preschoolers were significantly more likely to select the functional match over the object match in both the Causal Metaphor condition, *M* = 73.96%, *SE* = 5.32%, *t*(23) = 4.51, *p* < .001, and the Control Metaphor condition, *M* = 66.67%, *SE* = 5.14%, *t*(23) = 3.24, *p* = .004. Preschoolers’ ability to select the functional match over the object match in both conditions suggests that preschoolers already have some competence with relational reasoning.

#### Test Trials.

**Figure 2.**
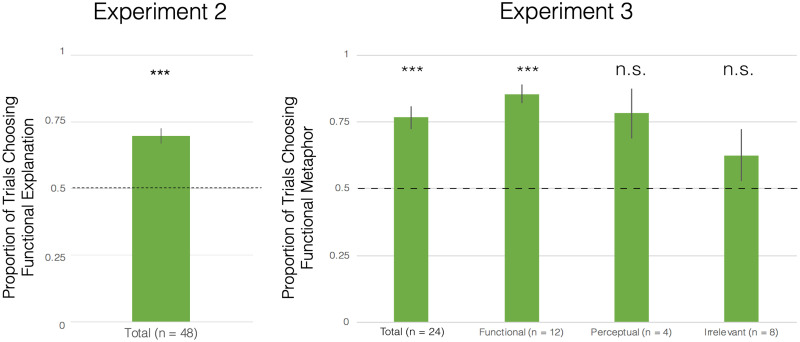
Test trial data from Experiment 2 and 3. Error bar shows 1 standard error from subject-level data.

Similar to the training trial results, there was also no significant difference in test trial performance between the Causal Metaphor condition and the Control Metaphor condition, *t*(46) = .54, *p* = .59. Consequently, we aggregated data across conditions and analyzed them together. We find that, when interpreting functional metaphors, preschoolers were significantly more likely to select functional explanations than perceptual explanations as the better interpretation of the functional metaphor, *M* = 69.79%, *SE* = 2.88%, *t*(47) = 6.87, *p* < .001, *d* = .99 (see Figure 2). Additionally, when examining individual participant responses, we find that none of the 48 preschoolers in the sample consistently preferred perceptual explanations (i.e., by selecting functional explanations on zero, one, or two of eight test trials). Rather, the individual participant responses ranged from a minimum of chance performance (i.e., selecting functional explanations on at least three out of eight test trials) to a maximum of consistent, unanimous preference for functional explanations (i.e., selecting functional explanations on eight out of eight test trials). Overall, the results of Experiment 2 suggest not only that preschoolers are capable of understanding the functional similarities between two objects in a functional metaphor, but also that preschoolers interpret functional metaphors based on functional similarities rather than perceptual similarities.

## EXPERIMENT 3

The results from Experiment 1A show that some preschoolers differentiate between functional metaphors and nonsense statements. The results from Experiment 2 suggest that this differentiation occurs because preschoolers are in fact capable of recognizing the abstract, functional similarities between objects in a functional metaphor. However, while the preschoolers in Experiment 1A were able to rate functional metaphors as “smarter” than nonsense statements, the overall sample of 128 preschoolers did not rate functional metaphors as “smart” above chance levels. Thus, in Experiment 3, we use another paradigm in order to provide converging evidence to support Experiment 1A’s claim that preschoolers are capable of differentiating between functional metaphors and nonsense statements. Specifically, in Experiment 3, we use a dichotomous choice task that directly contrasts functional metaphors against nonsense statements. In previous research (Vosniadou & Ortony, 1983), preschoolers presented dichotomous choice tasks that directly contrasted objects based on perceptual similarities against objects with no discernible similarity (e.g., “Is a sun like an orange or a chair?”) were able to select the perceptual match (e.g., the orange) over the nonsense match (e.g., the chair). While this previous research suggests an emerging competence with metaphors based on surface-level similarities such as color or shape, the present experiment is the first to explore this kind of dichotomous choice paradigm with functional metaphors based on more abstract, conceptual similarities.

### Methods

#### Participants.

We tested 24 participants in this study (*M* = 5.33 years; *SD* = .55 years; range = 4.11–5.95 years; 11 males, 13 females). One additional participant was tested but excluded due to fussiness. Children were recruited via e-mail from a local Bay Area child database and tested online over Zoom. All children viewed the stimuli using a computer or tablet. The experimenter asked parents to help standardize the experimental set-up by entering full-screen mode, hiding their own videos, and moving the experimenter’s video to the bottom-center of the screen.

#### Stimuli and Procedure.

The experimenter presented participants with the stimuli on a laptop computer. The experimenter introduced the study by saying, “We’re going to play with my friend Meg. Meg is going to ask questions. One person will give her an answer to her question. Then, another person will give her a different answer to her question. Your job is to point at the person who gives Meg the better answer. Let’s play!”

On each trial, Meg appeared on the screen and posed a question (e.g., “Can you tell me something about windows?”). Two people then appeared at the bottom of the screen. First, one person appeared on the left and provided a statement (e.g., a functional metaphor such as “Windows are eyes!”), as the two objects in the statement (e.g., a window and an eye) appeared on screen in a speech bubble. Then, another person appeared on the right and provided a statement (e.g., a nonsense statement such as “Windows are skirts!”), as the two objects in the statement (e.g., a window and a skirt) appeared on screen in a speech bubble. The experimenter prompted the participant to choose an explanation by asking, “Whose answer is better?” Once the participant provided a verbal response (e.g., “The person who said that windows are eyes”, “Eyes!”), the experimenter began the next trial. No feedback was provided. On the last trial, after the participant made a selection between the functional metaphor or the nonsense statement, the experimenter asked for an open-ended explanation about the similarity between the two components of whichever statement the participant chose (e.g., “How are roofs like hats? How are these two things alike?”).

There were eight test trials total, with each trial involving one of the eight functional metaphors from the test trials in Experiment 1. We counterbalanced whether the functional metaphor appeared on the left or the right. To better align Experiment 3’s paradigms with previous dichotomous-choice metaphor comprehension paradigms (e.g., contrasting functional and perceptual metaphors; Zhu et al., 2024), we asked children “Whose answer was better?” rather than “Whose answer was smarter?”

### Results & Discussion

#### Test Trials.

We first analyzed the data from the entire sample of preschoolers and found that overall, preschoolers are significantly above chance at selecting the functional metaphor over the nonsense statement, *M* = 76.56%, *SE* = 4.34%, *t*(23) = 6.12, *p* < .001, *d* = 1.25. This result suggests that preschoolers are not only capable of differentiating between functional metaphors and nonsense statements, but also prefer functional metaphors to nonsense statements.

#### Explanations.

As in Experiment 1A, we examined the explanations that preschoolers provided for how the two objects they selected on the last trial were alike (e.g., “How is a roof like a hat?”). There were 24 explanations total, as each child provided an explanation on the final trial. We used the same three explanation categories from Experiment 1A (functional, perceptual, and irrelevant) to code the explanations in Experiment 3. Fifty percent of preschoolers (12 out of 24) provided functional explanations that appealed to the function or structure of the two objects (e.g., “because you can see through them”, “because hat is on top of the head and roof is on top of the house”). Only 17% of preschoolers (4 out of 24) provided perceptual explanations that appealed to surface-level similarities between the two objects (e.g., “because they’re kind of shaped like rectangles”, “because they both look a little bit pointy”). Thirty-three percent of preschoolers (8 out of 24) provided irrelevant explanations (e.g., “I like strawberries and I like going to the pool”; “I don’t know why”). Two coders coded all explanations. Intercoder reliability was 83%, converging on the same category for 20 out of 24 explanations. The categorization of the remaining four explanations was resolved through discussion.

We analyzed data from the children who provided functional explanations, perceptual explanations, and irrelevant explanations separately, examining whether children from each explanation group were able to select the functional metaphors over the nonsense statements at significantly above-chance levels (see Figure 2). We find that the children who provided functional explanations (*n* = 12) were significantly more likely to select functional metaphors over nonsense statements, *M* = 85.42%, *SE* = 3.38%, *t*(11) = 10.47, *p* < .001. In contrast, children who provided perceptual explanations (*n* = 4) did not select functional metaphors over nonsense statements at above-chance levels, *M* = 78.13%, *SE* = 9.38%, *t*(3) = 3.00, *p* = .06. Similarly, children who provided irrelevant explanations (*n* = 8) also did not select functional metaphors over nonsense statements at above-chance levels, *M* = 62.5%, *SE* = 9.74%, *t* (7) = 1.28, *p* = .24.

Finally, we examined whether there were age differences across the three different explanation groups. A one-way between-subjects ANOVA found no effect between explanation type (Functional, Perceptual, Irrelevant) and age, *F*(2, 21) = 1.72, *p* = .20. Similarly, using Welch’s *t*-test to account for unequal variance due to the different sample sizes of the explanation groups, we find no difference in the ages of children across different explanation groups. Specifically, we find that the age of children who provided functional explanations (*M* = 5.53 years, *SE* = .12, range = 4.68–5.95 years) was not significantly different from the age of children who provided perceptual explanations (*M* = 5.11 years, *SE* = .40, range = 4.11–5.84 years), *t*(3.56) = 1.02, *p* = .37, or the age of children who provided irrelevant explanations (*M* = 5.14 years, *SE* = .20, range = 4.11–5.73 years), *t*(12.09) = 1.71, *p* = .11. Likewise, the age of children who provided perceptual explanations was also not signficantly different from the age of children who provided irrelevant explanations, *t*(4.54) = .06, *p* = .95. We also conducted Welch’s *t*-tests to examine whether the ages of any of the explanation groups was significantly different from the overall age of the entire sample of 24 children. We found no significant difference between the age of the children in the entire sample and the age of the children who provided functional explanations, *t*(28.30) = 1.23, *p* = .23, the age of the children who provided functional explanations, *t*(3.50) = 0.53, *p* = .63, and the age of the children who provided irrelevant explanations, *t*(11.89) = .85, *p* = .41. In summary, all of the age analyses found no age difference across the three types of explainers.

Thus, similar to the results in Experiment 1A, the overall significant result in Experiment 3 was primarily driven by a subset of children who explicitly provided functional explanations to justify their choices. In Experiment 1A, which presented functional metaphors and nonsense statements across trials, 25% of preschoolers in the sample were able to notice functional similarities and provide functional explanations. In Experiment 3, when presented a dichotomous choice task that explicitly contrasted functional metaphors against nonsense statements within trials, even more preschoolers (50% of the sample, as compared to 26% of the sample in Experiment 1) were able to notice the functional similarities and provide functional explanations.

#### Alternative Analyses.

Across all experiments, we generally found no differences in the significance or non-significance of results, between the subject-level frequentist analyses and the trial-level Bayesian analyses. However, a trial-level Bayesian logistic regression examining accuracy (i.e., proportion of functional metaphors selected) by explanation type (i.e., functional, perceptual, or irrelevant) with subject as a random intercept showed that children who provided functional explanations selected functional metaphors over nonsense statements, posterior mean accuracy = 87.52%, 95% credible interval [75.57%, 94.26%], probability that the estimate is above chance levels (log odds > 0) = 1.00, *and* children who provided perceptual explanations selected functional metaphors over nonsense statements, posterior mean accuracy = 80.51%, 95% credible interval [54.49%, 93.99%], probability that the estimate is above chance levels (log odds > 0) = .98. Children who provided irrelevant explanations did not have a preference for functional metaphors or nonsense statements, posterior mean accuracy = 64.47%, 95% credible interval [43.60%, 81.27%], probability that the estimate is above chance levels (log odds > 0) = .93. Consequently, while frequentist subject-level analyses suggested that only functional explainers selected functional metaphors over nonsense statements, trial-level Bayesian analyses suggest that both functional explainers and perceptual explainers selected functional metaphors over nonsense statements.

However, it is worth noting that the proportion of perceptual explainers in Experiment 3 was extremely small: only 4 out of 24 children provided perceptual explanations. Moreover, perceptual explainers must still use some form of relational reasoning to guide their choices in this task. Even if perceptual explainers do not notice the similarity between the two objects in the functional metaphors, they may notice the lack of similarity between the two objects in the nonsense statements, and use a mutual exclusivity strategy (e.g., Halberda, 2003) to guide their choices. Specifically, preschoolers in the perceptual explanation category may reason that objects in nonsense statements, such as “tires are paintbrushes”, have absolutely no similarity, and thus that the functional metaphors, such as “tires are shoes”, must be the better choice, even if they do not notice the functional similarity between the two objects in the functional metaphor. Thus, even the small proportion of perceptual explainers in Experiment 3 demonstrate some capacity for relational reasoning, and overall, Experiment 3 shows that preschoolers are capable of differentiating between functional metaphors and nonsense statements.

## GENERAL DISCUSSION

This paper introduces novel experimental paradigms that investigate preschoolers’ capacity to reason about *functional metaphors* – metaphors that draw equivalences based on abstract, relational, or structural commonalities – in an adult-like fashion. In contrast to prior work, our paradigms test children’s metaphor comprehension by testing their ability to discriminate functional metaphors from *nonsense statements*. Overall, these findings suggest that preschoolers indeed distinguish functional metaphors from nonsense statements. In particular, they seem to do this by noting the functional similarities between objects in functional metaphors. In Experiment 1A, preschoolers rated functional metaphors (e.g., “tires are shoes”) as “smarter” than nonsense statements (e.g., “boats are skirts”) in a “smart” or “silly” metalinguistic judgment paradigm. In Experiment 2, preschoolers preferred functional explanations (e.g., “both give water”) over perceptual explanations (e.g., “both are fluffy”) when interpreting the functional metaphors (e.g., “clouds are sponges”) used in Experiment 1. In Experiment 3, preschoolers preferred functional metaphors (e.g., “roofs are hats”) over nonsense statements (e.g., “roofs are scissors”) in a dichotomous-choice preference paradigm. Taken together, these three experiments demonstrate that children possess the capacity to understand metaphors based on abstract similarities at a much earlier developmental timepoint than previously assumed (Demorest et al., 1983; Silberstein et al., 1982; Winner et al., 1980).

Additionally, preschoolers not only selected functional explanations to interpret functional metaphors in Experiment 2; over a quarter of preschoolers in Experiment 1A and half of preschoolers in Experiment 3 could also explicitly articulate the functional similarities between two objects (e.g., “the hat shades you and the top of the roof does too”; “you can drive with wheels and walk with feet”), and the performance of these subsets of children drove the success of the entire sample in both studies. Preschoolers’ sophisticated functional explanations are consistent with previous work showing that preschoolers are capable of reasoning about abstract relations (Christie & Gentner, 2014; Goddu et al., 2020; Hochmann et al., 2017) and the functions of objects (Diesendruck et al., 2003; Haward et al., 2018). While it is striking that a single explanation measure can capture such meaningful variation in children’s overall performance on a task, these findings are consistent with other results showing that the one or two explanations provided by children at the end of a task can be meaningfully related to children’s reasoning at the overall task (e.g., Hochmann et al., 2017; Zhu & Gopnik, 2023, 2024). This relation has been found in paradigms relating both to children’s non-literal language comprehension (Zhu & Gopnik, 2024) and relational reasoning abilities (Hochmann et al., 2017). Moreover, while researchers previously thought that preschoolers might struggle to form metalinguistic judgments about metaphors (Pouscoulous & Tomasello, 2020), Experiment 1 shows that preschoolers can differentiate between abstract, functional metaphors and nonsense statements in a “smart” or “silly” metalinguistic judgment paradigm.

In contrast to previous research demonstrating a facilitative effect of causal framing on early relational reasoning (Goddu et al., 2020; Walker et al., 2016; Walker & Gopnik, 2017), we did not find such an effect on preschoolers’ performance in metaphor comprehension tasks in Experiment 1A and 2. In both Experiment 1A and 2, the experimenter explicitly introduced the test trials as a “new game” distinct from the causal training trials, and the format and stimuli used in the test trials were distinct from those used in the causal training trials. It is possible that these current methodological choices inadvertently encouraged children *not* to generalize a causal mindset that promotes relational thinking from the training trials to the test trials. Future work might investigate the kinds of contexts, and methodological choices, that facilitate or impede the transfer of causal reasoning capacities. Moreover, this lack of effect may be due to the fact that preschoolers were already surprisingly successful at multiple metaphor comprehension tasks without any kind of training. Specifically, in Experiment 1A, preschoolers in the baseline condition without training trials performed as well in the metaphor comprehension test trials as preschoolers in the three other conditions with training trials. Moreover, in Experiment 3, preschoolers successfully differentiated between functional metaphors and nonsense statements without any kind of training or warm-up. Thus, preschoolers were able to spontaneously apply relational reasoning skills in multiple metaphor comprehension tasks, and consequently did not require any kind of training – causal or otherwise – to elicit a “relational mindset” (e.g., Goldwater & Jamrozik, 2019; Simms & Richland, 2019). Indeed, preschoolers’ ability to spontaneously apply relational reasoning skills to multiple metaphor comprehension tasks once again suggest that preschoolers’ competence with abstract metaphors and relational reasoning may have been underestimated previously. With our novel paradigms, we were able to demonstrate preschoolers’ early ability to understand functional metaphors.

In addition to demonstrating preschoolers’ competence with metaphors, our paradigms find that preschoolers do not have more difficulty interpreting non-literal language, such as metaphors, than literal language, such as similes. Specifically, we find no difference between preschoolers’ performance when presented with metaphors (e.g., “Clouds are sponges”) or similes (e.g., “Clouds are *like* sponges”) in Experiment 1A. Indeed, the preschoolers in Experiment 1A were typically more flexible and accepting of non-literal language than adults in Experiment 1B, who seemed to rate metaphors as “sillier” than similes. While adults seemed to show a preference for novel linguistic comparisons in simile rather than metaphor form (Bowdle & Gentner, 2005), children have not developed this preference, and perform equally well with metaphors and similes. Moreover, preschoolers performed successfully in Experiments 2 and 3, which were conducted solely with metaphors as opposed to similes. Consequently, the results of the three current experiments converge with other findings suggesting that preschoolers do not have difficulty with some kinds of non-literal language (e.g., Zhu, 2021; Zhu & Gopnik, 2024). Moreover, in the broader literature on language acquisition, metaphor is often discussed as a case study of pragmatic reasoning (Gibbs, 2023; Goodman & Frank, 2016; Kao et al., 2014; Tonini et al., 2023). Consequently, the present research demonstrating children’s early emerging metaphor comprehension also converges with recent work showing that children may acquire competence in a range of other pragmatic inferences, such as presuppositions (Domaneschi et al., 2022; Pouscoulous, 2023) and scalar implicatures (Foppolo et al., 2012; Horowitz et al., 2018), earlier than previously expected.

How might we reconcile the current results, which demonstrate that preschoolers successfully understand abstract, functional metaphors, with earlier work demonstrating failures in metaphor comprehension amongst young children (Demorest et al., 1983; Silberstein et al., 1982; Winner et al., 1976, 1980)? One possibility is that the current research used novel paradigms that are more sensitive measures of children’s linguistic capacities. For example, previous paradigms often juxtapose functional metaphors against perceptual metaphors, and thus pose additional challenges beyond comprehension. Specifically, since functional metaphors and perceptual metaphors may both be technically true and acceptable, children may *understand* both kinds of metaphors, but *select* one metaphor over another on the basis of some other dimension, such as informativeness or usefulness. Thus, paradigms juxtaposing abstract metaphors and perceptual metaphors may be an especially difficult test of metaphor comprehension. In contrast, our new paradigms ask children to evaluate metaphors and nonsense statements individually (Experiment 1), or juxtapose abstract metaphors against nonsense statements (Experiment 3). Preschoolers may be better able to demonstrate their comprehension of abstract, functional metaphors in the absence of equally acceptable perceptual metaphors. Indeed, developmental psychologists often find that children succeed in cognitively complex tasks earlier in development when using more sensitive experimental paradigms. For example, in the literature on children’s analogical reasoning abilities, children do not succeed at classic experimental paradigms of analogical reasoning until four or five years of age (Hochmann et al., 2017), but succeed at novel and more sensitive experimental paradigms of analogical reasoning years earlier in development (Walker et al., 2016; Walker & Gopnik, 2017).

These positive findings on metaphor comprehension pave the way for new and exciting future research directions. For example, while the current research focuses on the success of the overall sample of preschoolers, the results of Experiment 1A and 3 show that the success of the overall group is driven by a subset of preschoolers who can explicitly provide functional explanations. One interesting future direction might be to explore why some preschoolers, but not others, provide functional explanations and understand functional metaphors. For example, perhaps the preschoolers providing explicit explanations have better relational reasoning skills, more conceptual knowledge about the items in the metaphors (Keil, 1986), or better executive function abilities (e.g., Carriedo et al., 2016). Indeed, research on individual differences in relational reasoning and metaphor comprehension in adolescents and adults suggests that individual differences in executive function (e.g., working memory capacity) may be related to metaphor comprehension (Carriedo et al., 2016; Grossnickle et al., 2016; Kazmerski et al., 2003), but it is unknown whether there is also a positive relation between executive function and metaphor comprehension in young children. Thus, while relational reasoning plays a crucial role in metaphor comprehension (Bowdle & Gentner, 2005; Gentner, 1988; Gentner & Clement, 1988; Holyoak, 2019), follow-up studies exploring individual differences might shed light on how other cognitive mechanisms, such as conceptual knowledge and executive function, also contribute to children’s capacity to provide functional explanations and understand functional metaphors.

Additionally, while the present findings demonstrate that preschoolers can differentiate functional metaphors from nonsense statements, future research could explore whether preschoolers understand metaphors presented in other, more naturalistic settings. Experimental studies on children’s metaphor comprehension frequently juxtapose multiple metaphors against each other (e.g., Silberstein et al., 1982; Vosniadou & Ortony, 1983), but more naturalistic contexts such as parent-child conversations or written poetry might focus on a single metaphor at a time. Thus, while Experiment 1A and 3 use novel paradigms involving both functional metaphors and nonsense statements, future work might investigate whether preschoolers also understand metaphors under different circumstances, without the direct juxtaposition of nonsense statements.

Another exciting potential research question is whether preschoolers are capable of using metaphor and relational reasoning in the service of other complex learning processes, such as thinking and reasoning about abstract concepts in the contexts of scientific discovery (Kuhn, 1993) and conceptual change (Xu, 2019). Researchers have argued that linguistic metaphors provide useful conceptual frameworks, allowing new, insightful ways of reasoning about old concepts (Thibodeau et al., 2017), as well as facilitating the acquisition of novel concepts and word meanings (Bowdle & Gentner, 2005; Holyoak & Stamenkovíc, 2018). Thus, metaphors could potentially be powerful tools for children, helping them acquire more information about the world, Indeed, recent work demonstrates that children can learn from metaphors (Zhu & Gopnik, 2023), though future research could replicate and extend these initial findings on how children might leverage metaphors for further thinking and reasoning.

While our work presents interesting new evidence on preschoolers’ metaphor comprehension abilities, the present work also has limitations that warrant further investigation. For example, Experiment 1 demonstrates that preschoolers and adults differentiate between functional metaphors and nonsense statements when asked to rate these utterances as “smart” or “silly” in a metalinguistic judgment paradigm. However, it is not entirely clear how preschoolers and adults interpret the meanings of the words “smart” and “silly” in this context. Experiment 1A’s preschooler explanation data suggest that preschoolers’ “smart” and “silly” judgments are picking up some kind of meaningful signal, since the subset of preschoolers who provided functional explanations also rated functional metaphors as “smarter” than nonsense statements. However, even some adults tended to judge both functional metaphors and nonsense statements as “silly”, suggesting that the current paradigm may sometimes be confusing for adults, and thus likely sometimes confusing for children too. Consequently, more empirical work is required to validate and potentially improve our novel paradigm. Since there was no difference between preschoolers’ performance on the Causal Metaphor and Causal Simile conditions in Experiment 1, we then focused on running alternate paradigms (e.g., Experiments 2 and 3) that could provide converging evidence for children’s metaphor comprehension abilities. However, future work could run additional metaphor conditions in the “smart” and “silly” metalinguistic judgment paradigm (e.g., a metaphor condition with non-causal training, or perhaps even a metaphor condition with no training in order to demonstrate spontaneous success) to replicate and extend the current findings. Moreover, one way to validate the paradigm is to investigate how children respond when presented with other kinds of utterances, such as perceptual metaphors used in the previous literature (e.g., “Tires are donuts”; Zhu et al., 2024) or category statements which are literally true or false (e.g., “Dalmatians are dogs”; “Dalmatians are fish”). Future research may also explore another variation of this paradigm, by examining whether adults and children can provide “true” or “false” judgments rather than “smart” or “silly” judgments, and whether true/false judgments may be less noisy than smart/silly judgments.

Another limitation is that there may be potential difficulties drawing conclusions from Experiment 3, in which preschoolers selected functional metaphors over nonsense statements. While preschoolers may have selected functional metaphors because they understood the analogies underlying the functional metaphors, a deflationary alternative is that children used a mutual exclusivity strategy (Halberda, 2003) – that is, preschoolers rejected the nonsense statements without understanding the functional metaphors. Given that half of the children in Experiment 3 provided functional explanations to justify their responses, we believe that at least some of the children are not using a mutual exclusivity strategy, but rather understanding the abstract functional similarities underlying the functional metaphors. However, we note that the conclusions drawn from Experiment 3 are tentative, and that future research should look for additional, stronger evidence that children understand functional metaphors in the absence of training phases with feedback (as in Experiments 1 and 2) or juxtapositions with nonsense statements (as in Experiment 3).

Moreover, additional work with non-Western populations is required to determine the generalizability of the current findings. Given the evidence of cross-cultural variation in the development of relational reasoning abilities (Carstensen et al., 2019), it is possible that children in other cultures may also understand metaphors sooner or later in development than U.S. children. Another limitation of our current work is its reliance on WEIRD (i.e., Western, educated, industrialized, rich, and Democratic; Henrich et al., 2010) convenience samples; thus, future work should investigate the possibility of early cross-cultural diversity in children’s metaphor comprehension abilities.

Overall, the current research shows that 4- to 5-year-olds are already capable of understanding functional metaphors based on abstract similarities between two disparate concepts. Preschoolers’ success with functional metaphors provides exciting groundwork for future research on children’s early comprehension of non-literal language, and how children think, reason, and learn more broadly.

## ACKNOWLEDGMENTS

We are grateful to members of the Cognitive Development and Learning Lab at UC Berkeley, especially Sophie McMullen, Zhimeng Li, Emily Demsetz, and Esmerelda Herrera for their help with data collection. Thanks also to the Lawrence Hall of Science, Children’s Creativity Museum, and the preschools, parents, and children who made this research possible.

## FUNDING INFORMATION

This work was supported by an NSERC Post-Graduate Doctoral Fellowship to RZ [532517-2019].

## AUTHOR CONTRIBUTIONS

R.Z.: Conceptualization; Data curation; Formal analysis; Funding acquisition; Investigation; Methodology; Project administration; Writing – original draft; Writing – review & editing. M.K.G.: Conceptualization; Methodology; Writing – review & editing. L.Z.Z.: Data curation; Formal analysis; Writing – review & editing. A.G.: Conceptualization; Investigation; Methodology; Project administration; Supervision; Writing – review & editing.

## DATA AVAILABILITY STATEMENT

Deidentified data and analysis scripts have been made publicly available via OSF and can be accessed at https://osf.io/cpk92. The studies reported in this paper were not preregistered. The materials used in this study are available on request.
